# Resatorvid alleviates experimental inflammatory TMJOA by restraining chondrocyte pyroptosis and synovial inflammation

**DOI:** 10.1186/s13075-023-03214-4

**Published:** 2023-11-29

**Authors:** Xin Liu, Huimin Li, Yaping Feng, Huilin Guo, Yingjie Li, Jin Ke, Xing Long

**Affiliations:** 1 State Key Laboratory of Oral & Maxillofacial Reconstruction and Regeneration, Key Laboratory of Oral Biomedicine Ministry of Education, Hubei Key Laboratory of Stomatology, School & Hospital of Stomatology, Wuhan University, 237 Luoyu Road, Wuhan, 430079 Hubei China; 2 Department of Oral and Maxillofacial Surgery, School & Hospital of Stomatology, Wuhan University, Wuhan, Hubei China

**Keywords:** TMJOA, Resatorvid, Chondrocyte pyroptosis, Macrophage inflammation, NLRP3, ROS

## Abstract

**Objectives:**

Innate immunity plays a significant role in the pathogenesis of temporomandibular joint osteoarthritis (TMJOA), which is characterized by synovial inflammation and condylar cartilage degradation. We are urged to investigate the impact of Resatorvid, a preventative drug that inhibits Toll-like receptor 4 (TLR4), on experimental inflammatory TMJOA pathology.

**Methods:**

An intra-articular injection of complete Freund’s adjuvant (CFA) was used to induce an experimental inflammatory mouse TMJOA model, and TLR4 expression was identified by immunofluorescent labeling. Intraperitoneal injections of Resatorvid were administered to CFA-induced TMJOA mice, and the pathology of TMJOA animals with and without Resatorvid treatment was examined by H&E, Safranin-O/Fast Green, and TRAP staining, as well as micro-CT, immunohistochemistry, and immunofluorescence. The impact of Resatorvid on chondrocyte pyroptosis and macrophage inflammation was further investigated using ATDC5 chondrocytes and RAW264.7 macrophages pretreated with relevant antagonists.

**Results:**

CFA-induced TMJOA mice revealed remarkable synovial inflammation, together with a time course of cartilage degradation and bone destruction, with TLR4 elevated in the synovium and condylar cartilage. Prophylactic treatment with Resatorvid mitigated synovial inflammation, cartilage degeneration, and bone destruction in CFA-induced TMJOA mice and downregulated MyD88/NF-κB expression. Ex vivo studies demonstrated that Resatorvid treatment alleviated NOD-like receptor protein 3 (NLRP3)-mediated chondrocyte pyroptosis and degeneration and relieved macrophage inflammation by preventing reactive oxygen species (ROS) production through NLRP3 signaling.

**Conclusion:**

Prophylactic treatment with Resatorvid alleviates TMJOA pathology by inhibiting chondrocyte pyroptosis and degeneration, as well as ROS-induced macrophage inflammation, through TLR4/MyD88/NF-κB/NLRP3.

**Supplementary Information:**

The online version contains supplementary material available at 10.1186/s13075-023-03214-4.

## Introduction

Progressive synovial inflammation, condylar cartilage degradation, and bone destruction have been accepted as three vital pathological characteristics of temporomandibular joint osteoarthritis (TMJOA), a degenerative joint disease without radical therapeutic therapies [1, 2]. TMJOA is a persistent pathological state, causing joint tenderness, crepitus, noises, and even restricted mouth opening, and it brings great trouble to patients’ lives. As one of the most serious types of temporomandibular disorder (TMD), the prevalence of TMJOA ranges from 18.01 to 84.47% in TMD patients and affects 8–16% of the population worldwide, while its exact etiological mechanism is still not completely known [3, 4].

Pattern recognition receptors (PRRs) were confirmed to participate in the pathogenesis of osteoarthritis (OA), including synovial inflammation and cartilage degradation [5]. Resatorvid (TAK-242) is a novel chemopreventive agent that suppresses the production of inflammatory mediators during innate immune reactions by restraining toll-like receptor 4 (TLR4), a classical membrane PRR [6]. A previous study confirmed that the excitation of TLR4 contributes to the proinflammatory activation of cartilage degradation and macrophage polarization during OA [7], emphasizing the involvement of inflammatory responses triggered by the activation of innate immune receptors over OA progression [8]. However, there are few studies concerning the regulatory effect of TLR4 on the pathological process of TMJOA to date. Specifically, the regulatory role of TLR4 and Resatorvid in inflammation-irritated TMJOA pathology is still unknown.

Condylar cartilage consists of chondrocytes and the surrounding environment (extracellular matrix, ECM). Degradation of ECM irritated by chondrocyte degeneration during TMJOA not only changes the properties of cartilage but also affects the phenotype of chondrocytes [9]. Moreover, chondrocyte death, including apoptosis, autophagy, pyroptosis, and ferroptosis, accelerates the destruction of articular cartilage. Pyroptosis, a gasdermin-induced necrotic cell death featuring secretion of pro-inflammatory cytokines and a lytic form of cell death, has become widely known [10, 11]. Although NOD-like receptor pyrin 3 (NLRP3) inflammasome-mediated pyroptosis has been confirmed to participate in the synovitis of TMJOA, little is known about the role of chondrocyte pyroptosis in condylar cartilage degeneration during TMJOA progression [12].

Macrophages and fibroblasts are two crucial cell components of synovial tissues. Increasing evidence suggests that macrophages play a vital role in modulating synovial inflammation and thus OA severity via the secretion of various proinflammatory cytokines [13, 14]. Inflammation is closely related to cellular oxidative stress, which causes the release of reactive oxygen species (ROS), thereby accelerating inflammatory responses [15, 16]. However, the involvement of ROS in synovial inflammation in TMJOA is inadequately understood.

The NLRP3 inflammasome is a cytoplasmic supramolecular complex that is activated in response to cellular perturbations triggered by inflammation [17]. NLRP3 contributes to gasdermin D (GSDMD)-mediated pyroptosis, and cumulative evidence has confirmed the involvement of NLRP3 in ROS-induced oxidative stress, suggesting the vital role of NLRP3 in pyroptosis and oxidative stress under inflammatory responses [18, 19]. Considering the vital effect of both inflammation and innate immune reactions during OA, we speculate that Resatorvid regulates NLRP3-mediated condylar chondrocyte pyroptosis and synovial macrophage oxidative stress during TMJOA pathology.

Herein, intraperitoneal administration of Resatorvid was utilized to explore the effect of TLR4 in complete Freund’s adjuvant (CFA)-induced TMJOA in mice, a highly inflammatory animal model [20]. Synovial inflammation, cartilage degeneration, bone destruction, and the expression of classical downstream signals of TLR4 (MyD88 and NF-κB) [21, 22] were compared between CFA-induced TMJOA mice, with and without Resatorvid treatment. Moreover, the effect of Resatorvid on chondrocyte pyroptosis and macrophage oxidative stress was analyzed using ATDC5 chondrogenic cells and Raw264.7 macrophage culture.

## Materials and methods

### An inflammatory TMJOA model

The protocols for animal studies were approved by the Ethics Committee for Animal Research, School and Hospital of Stomatology, Wuhan University (protocol No. S07918060A), and in accordance with the National Research Council’s Guide for the Care and Use of Laboratory Animals. A CFA-induced mouse TMJOA model was employed in the present study, with 27 male C57BL/6 J mice (aged 8–10 weeks, weighing 20–25 g) purchased from the Experimental Animal Centre of Hubei Province. Mice were fed a standard diet and housed in pathogen-free cages with 12 h circadian rhythms and randomly divided into saline, CFA, and CFA + Resatorvid groups, with each group containing 9 mice. CFA-induced TMJOA mice were elicited by bilateral intra-articular injections of 20 μL CFA (Sigma, F5881) using an anterosuperior puncture technique as previously described [23]. The saline group received bilateral intra-articular injections of isopyknic saline. Mice in the saline, CFA, and CFA + Resatorvid groups were analyzed at 1, 2, and 4 weeks after intra-articular injection, with 3 mice per time point (6 joints, *n* = 6).

### Administration of Resatorvid

Resatorvid (HY-11109, MCE), a specific TLR4 antagonist interfering with the interactions between TLR4 and its intracellular adaptors, was employed in the present study. The CFA + Resatorvid group was treated with intraperitoneal injections of Resatorvid at a dose of 10 mg/kg [24] 1 day before intra-articular CFA induction and maintained twice a week post intra-articular injection.

### Micro-CT analysis

Collected temporomandibular joint (TMJ) tissues from each group were fixed in 4% paraformaldehyde solution for 24 h and flushed overnight. Afterwards, TMJ specimens used for bone morphological analysis were scanned by micro-CT (filter Al 0.2 mm, 50 kV, 500 μA, 12.59 μm, SkyScan1176) to record the change in subchondral bone. The scanned data were reconstructed by NRecon, with sagittal sections processed with CTAn. Furthermore, 3D images were dimensionally reconstructed by CTvox for morphological assessment.

### Histological detection

After paraformaldehyde fixation and ethylene diamine tetra-acetic acid (EDTA) decalcification, all TMJ specimens were subjected to gradient dehydration and embedded in paraffin. Then, the embedded specimens were cut into continuous sagittal sections, each at 5 μm. After dewaxing in xylene and gradient hydration, H&E, Safranin-O/Fast Green, and Masson staining were performed according to the manufacturer’s protocols to examine synovial inflammation, cartilage degeneration, and bone destruction, respectively. H&E staining of synovial tissues was quantified by the number of synovial lining layers. Cartilage degradation was evaluated by condylar cartilage thickness of H&E staining and modified Mankin OA score of Safranin-O/Fast Green staining, on the basis of predecessors’ methods [25]. The degeneration of subchondral bone was measured by the percentage of unmineralized bone based on Masson staining, which averaged from anterior, middle and posterior, 3 selected areas of the subchondral bone. Moreover, TRAP staining was utilized to detect osteoclast activity within the subchondral bone, with the number of TRAP-positive cells used for quantification.

### Immunohistochemistry and immunofluorescence

Immunohistochemistry and immunofluorescence of tissue slices were performed as described previously [26], with primary antibodies utilized as follows: rabbit anti-TLR4 (1:400, 19811–1-AP, Proteintech), rabbit anti-CD34 (1:300, GB111693, Servicebio), rabbit anti-F4/80 (1:100, GB113373, Servicebio), rabbit anti-IL-1β (1:200, ab9722, Abcam), rabbit anti-TNF-α (1:200, ab6671, Abcam), rabbit anti-iNOS (1:100, GB11119, Servicebio), rabbit anti-COX-2 (1:300, 12375–1-AP, Proteintech), rabbit anti-ADAMTS5 (1:200, ab41037, Abcam), rabbit anti-MMP13 (1:200, 18165–1-AP, Proteintech), rabbit anti-Caspase1 (1:200, BA2220, Boster), rabbit anti-GSDMD (1:200, ab219800, Abcam), rabbit anti-Aggrecan (1:600, 13880–1-AP, Proteintech), rabbit anti-MyD88 (1:300, 23230–1-AP, Proteintech), rabbit anti-NF-κB p65 (1:300, 10745–1-AP, Proteintech), and rabbit anti-NLRP3 (1:300, 19771–1-AP, Proteintech). The positive expression of condylar cartilage and synovial tissues was quantified by the rate of positive cells, average optical density (AOD), or integral optical density (IOD) of positive staining, respectively.

### Chondrocyte and macrophage culture

Mouse ATDC5 chondrogenic cells (BFB Biotechnology Development Co., LTD) were cultured in DMEM/F-12 and induced by insulin-transferrin-selenium (ITS) as previously mentioned [2]. After chondrogenic induction, ATDC5 cells were stimulated with lipopolysaccharide (LPS) at a dose-course for TLR4 activation. Resatorvid (10 μM, HY-11109, MCE), PDTC (50 μM, HY-18738, MCE), and MCC950 (10 μM, HY-12815A, MCE) were added to ATDC5 cells before IL-1β (10 ng/ml, 200-01B, PeproTech) incubation. Moreover, ATDC5 cells were transfected with NLRP3 lentiviruses (Lv-NLRP3, MOI = 10) from Genechem Co., Ltd. for NLRP3 overexpression.

RAW264.7 macrophages (Procell Life Science & Technology Co., Ltd.) were cultured in DMEM with 10% fetal bovine serum (FBS) and incubated with LPS (100 ng/ml, L4391, Sigma) combined with IFN-γ (20 ng/ml, CM41, Novoprotein) for M1 phenotype polarization. Pretreatment with Resatorvid (10 μM), MCC950 (10 μM) and N-acetylcysteine (NAC, 10 mM, HY-B0215, MCE) was utilized to analyze the effect of TLR4, NLRP3, or ROS suppression on the inflammatory responses of M1 macrophages. Furthermore, M1 macrophages were enriched in ROS by incubation with H_2_O_2_ (100 μM, 323381, Sigma) for 24 h. Each experiment was repeated thrice independently.

### ELISA analysis

One day following phenotype induction and mediator administration, RAW264.7 macrophages were replaced with fresh medium and collected 24 h after culture. After centrifugation to remove precipitation, the concentrations of IL-1β, TNF-α, and IL-6 in macrophage supernatants were detected by ELISA analysis. The experimental procedure of ELISA analysis was performed based on the manufacturer’s protocols, including a mouse IL-1β ELISA kit (RX203063M, Ruixin bio, Quanzhou, China), mouse TNF-α ELISA kit (JL10484, Jianglai bio, Shanghai, China), and mouse IL-6 ELISA kit (EMC004, NeoBioscience, Shenzhen, China). Each experiment was independently repeated thrice.

### Immunocytofluorescence

RAW264.7 macrophages and ATDC5 cells used for immunocytofluorescence detection were permeabilized with 0.3% Triton X-100 after 4% paraformaldehyde fixation. Afterwards, the cells were incubated with 2.5% bovine serum albumin (BSA) for antigen blocking and treated with primary antibody against NLRP3 (1:300, 19771–1-AP, Proteintech) overnight at 4 °C. Subsequently, Dylight 488, goat anti-rabbit IgG or DyLight 594, and goat anti-rabbit IgG were added and reacted at 37 °C for 60 min, and the nuclei were stained with 4′,6-diamidino-2-phenylindole (DAPI). Positive reactivity of cells was captured under a fluorescence microscope and quantified by the value of AOD.

### Pyroptosis detection and ROS measurement

To morphologically analyze chondrocyte pyroptosis, ATDC5 cells were cultured to 80% confluence and then treated with Hoechst staining combined with propidium iodide (PI) staining, according to previous reports [27]. Briefly, cells were stained with PI for 15 min at 37 °C, and nuclei were stained with Hoechst 33342. Lytic cell death was visualized and measured by PI incorporation to label dying cells. Fluorescence images were captured using a fluorescence microscope, and cellular morphology was observed through brightfield images.

For ROS measurement, RAW264.7 macrophages were labeled with cell-permeable dihydroethidium (DHE) fluorogenic probes for 30 min at 37 °C, followed by nuclear staining with DAPI. The released ROS from macrophages were detected by fluorescence microscopy under an excitation wavelength of 594 nm and quantified by the AOD of positive labeling.

### Western blotting

After cells were lysed with RIPA buffer and centrifuged to separate sediment, the protein samples were loaded and separated by electrophoresis in a 10% SDS–PAGE gel. The proteins were transferred to a PVDF membrane and incubated with the following primary antibodies at 4 °C overnight after blocking with 5% skim milk: rabbit anti-TLR4 (1:1000, GB11519, Servicebio), rabbit anti-NLRP3 (1:2000, ab263899, Abcam), rabbit anti-ADAMTS5 (1:2000, ab41037, Abcam), rabbit anti-MMP13 (1:1000, GB11247-1, Servicebio), rabbit anti-Caspase1 (1:2000, BA2220, Boster), rabbit anti-GSDMD (1:1000, ab219800, Abcam), rabbit anti-COX-2 (1:1000, #12282, CST), mouse anti-iNOS (1:1000, MA5-17139, Invitrogen), rabbit anti-NF-κB (1:2000, 10745–1-AP, Proteintech), rabbit anti-IL-1β (1:6000, GB11113, Servicebio), rabbit anti-IL-6 (1:4000, GB11117, Servicebio), and mouse anti-GAPDH (1:5000, RAB0101, Frdbio). On the second day, the membranes were incubated with HRP-conjugated secondary antibodies at 37 °C for 1 h, and protein bands were visualized by a hypersensitive ECL kit (PMK003, BioPM). The expression of proteins was quantified and normalized to GAPDH using the ImageJ 1.8.0 software.

### Statistical analysis

Statistical analysis within this study was conducted by Prism 8.0 (GraphPad). Quantitative data are presented as the mean ± SEM, and *p* < 0.05 was considered statistically significant. Data between groups were analyzed by Student’s *t* test or one-way ANOVA followed by Dunnett’s multiple comparisons test, and two-way ANOVA followed by Sadak’s multiple comparisons test was used for group analysis. Beforehand, a Shapiro–Wilk test for normality was conducted to determine whether the data were parametric, with *F* tests utilized to check the homogeneity of variance.

## Results

### Elevation of TLR4 in the synovium and cartilage of CFA-induced TMJOA mice

**Fig. 1:**
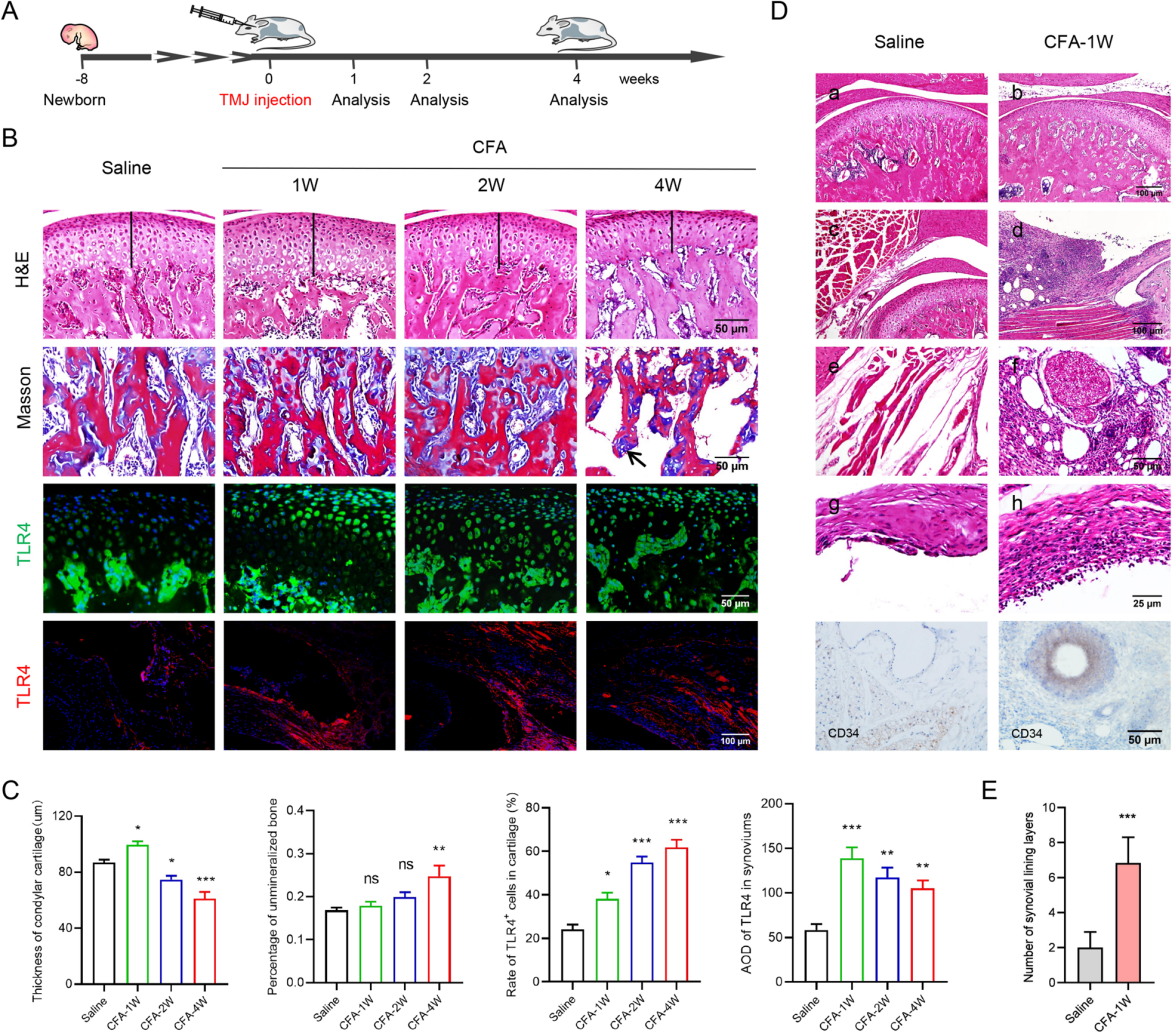
Histological characteristics and TLR4 expression in CFA-induced TMJOA mice. **A** Scheme regarding the establishment of TMJOA mice by bilateral intra-articular CFA injection and analyzed at 1, 2, and 4 weeks post-injection. **B**, **C** Representative images of H&E, Masson staining, and TLR4 immunofluorescence staining for TMJ tissue slices. **B** Quantitative analysis showed that there was a time course of condylar cartilage thinning and unmineralized bone increase 1, 2, and 4 weeks after CFA injection, with the expression of TLR4 enhanced in the condylar cartilage and synovium of CFA-induced TMJOA mice (**C**), one-way ANOVA, mean ± SEM, *n* = 6 (*p < 0.05, **p < 0.01, ***p < 0.001 vs saline group, ns indicates no significant difference). **D** H&E staining disclosed remarkable inflammatory exudation (b), inflammatory cell infiltration (d), lipid droplet and lymphoid follicle formation (f), and synovial lining layer thickening (h) in inflammatory synovial tissues of CFA-induced TMJOA mice at 1 week, accompanied by CD34 immunochemical staining in the synovial vascular region of TMJOA mice. **E** Quantitative analysis revealed that the number of synovial lining layers increased in CFA-induced TMJOA mice at 1 week, Student’s *t* test, mean ± SEM, *n* = 6 (***p < 0.001)

CFA-induced TMJOA mice were established by bilateral intra-articular injections of CFA and analyzed 1, 2, and 4 weeks post-injection (Fig. 1A). As depicted in Fig. 1B and C, there was a time-course of cartilage degeneration and bone destruction in CFA-induced TMJOA mice, accompanied by elevation of TLR4 in condylar cartilage and synovial tissues of TMJOA mice. Specifically, CFA-induced TMJOA mice at 4 weeks exhibited the most serious cartilage degradation and bone destruction, as well as the highest expression of TLR4 in cartilage, although cartilage was thickened temporally at 1 week (Fig. 1B and C). Inflammatory exudation, inflammatory cell infiltration, lipid droplets, lymphoid follicle formation, and synovial lining layer thickening were uncovered in inflammatory synovial tissues of TMJOA mice at 1 week, accompanied by remarkable CD34 staining in the vascular area of the synovium, compared with saline-injected mice (Fig. 1D and E). These results revealed the upregulation of TLR4 in inflammatory synovial tissues and degenerative condylar cartilage of CFA-induced TMJOA mice.

### Prophylactic treatment with Resatorvid alleviates the pathology of CFA-induced TMJOA mice

**Fig. 2:**
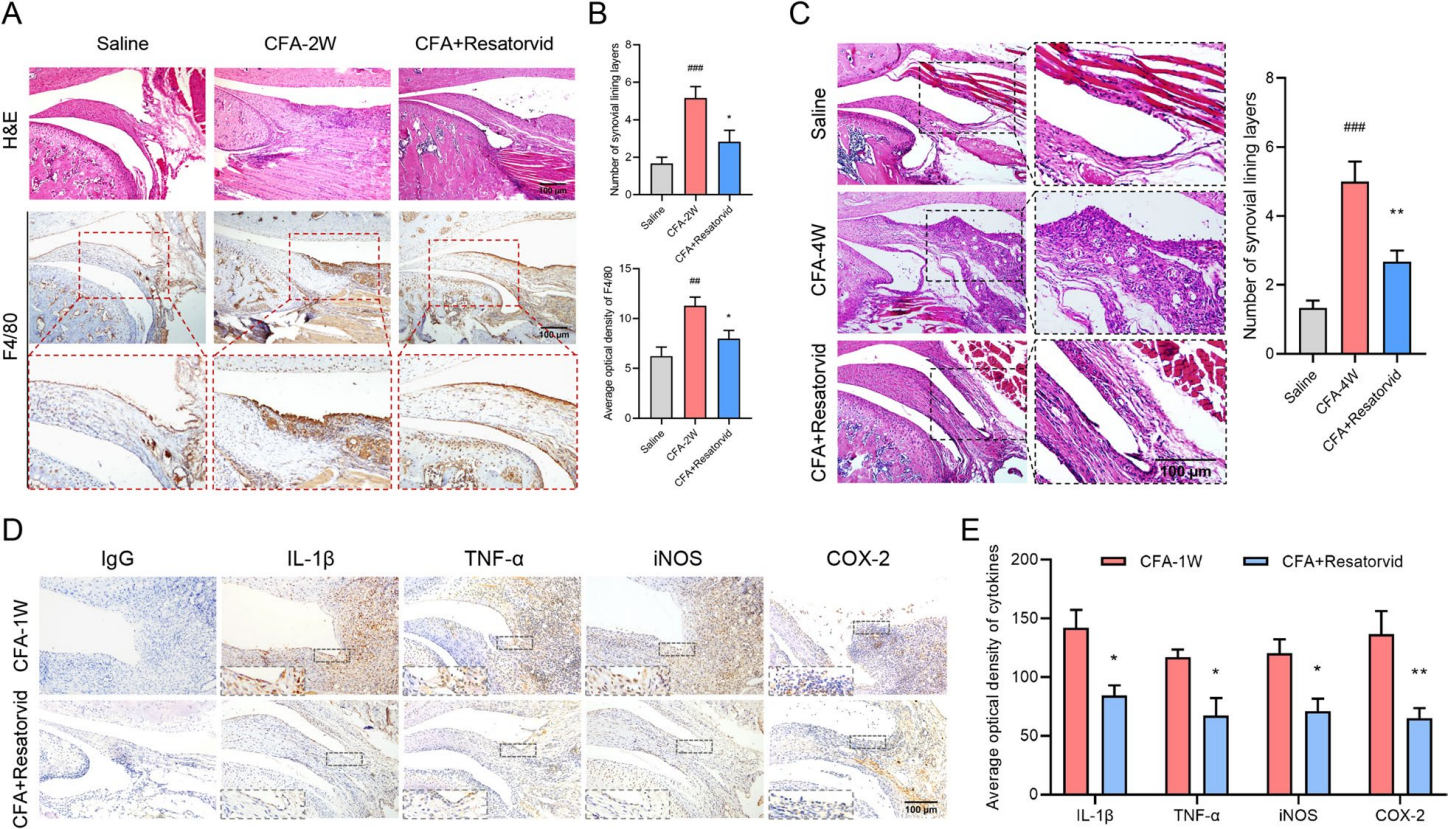
Systemic administration of Resatorvid relieves synovial inflammation in CFA-induced TMJOA mice. **A**, **B** Synovial inflammation in CFA-induced TMJOA mice was analyzed by H&E and F4/80 and immunohistochemical staining (**A**). Intraperitoneal administration of Resatorvid mitigated CFA-induced inflammatory hyperplasia and macrophage activation in the synovium of TMJOA mice at 2 weeks, and the number of synovial lining layers and the average optional density of F4/80 declined in CFA + Resatorvid-treated mice (**B**), one-way ANOVA, mean ± SEM, *n* = 6 (##*p* < 0.01, ###*p* < 0.001 vs saline group; *p < 0.05 vs CFA group). **C** Representative images of H&E staining showing that the activation of the synovium was relieved in Resatorvid-treated TMJOA mice, accompanied by thinning of the synovial lining layers, one-way ANOVA, mean ± SEM, *n* = 6 (###*p* < 0.001 *vs* saline group; **p < 0.01 vs CFA group). **D**, **E** Immunohistochemical staining detecting the expression of proinflammatory cytokines (IL-1β, TNF-α, iNOS, and COX-2) in the synovial tissues of CFA group mice and CFA + Resatorvid group mice (**D**). Quantitative analysis showed that the average optical density of cytokines above decreased in Resatorvid-treated TMJOA mice compared to CFA group mice at 1 week (**E**), Student’s *t* test, mean ± SEM, *n* = 5 (*p < 0.05, **p < 0.01 vs CFA group)

Synovial inflammation in CFA-induced TMJOA mice was analyzed by H&E and immunohistochemical staining. As shown in Fig. 2A and B, conspicuous synovial inflammatory hyperplasia and macrophage biomarker F4/80 immunohistochemical staining were exhibited in CFA-induced TMJOA mice at 2 weeks, while administration of Resatorvid alleviated these phenomena. Similar results were found in TMJOA mice at 4 weeks, and intraperitoneal injections of Resatorvid downregulated the incrassation of synovial lining layers in CFA-induced TMJOA mice (Fig. 2C). Moreover, immunohistochemical analysis showed that the expression of proinflammatory cytokines (IL-1β, TNF-α, iNOS, and COX-2) in synovial tissues was restrained in Resatorvid-treated TMJOA mice compared with CFA-induced TMJOA mice (Fig. 2D and E).

**Fig. 3:**
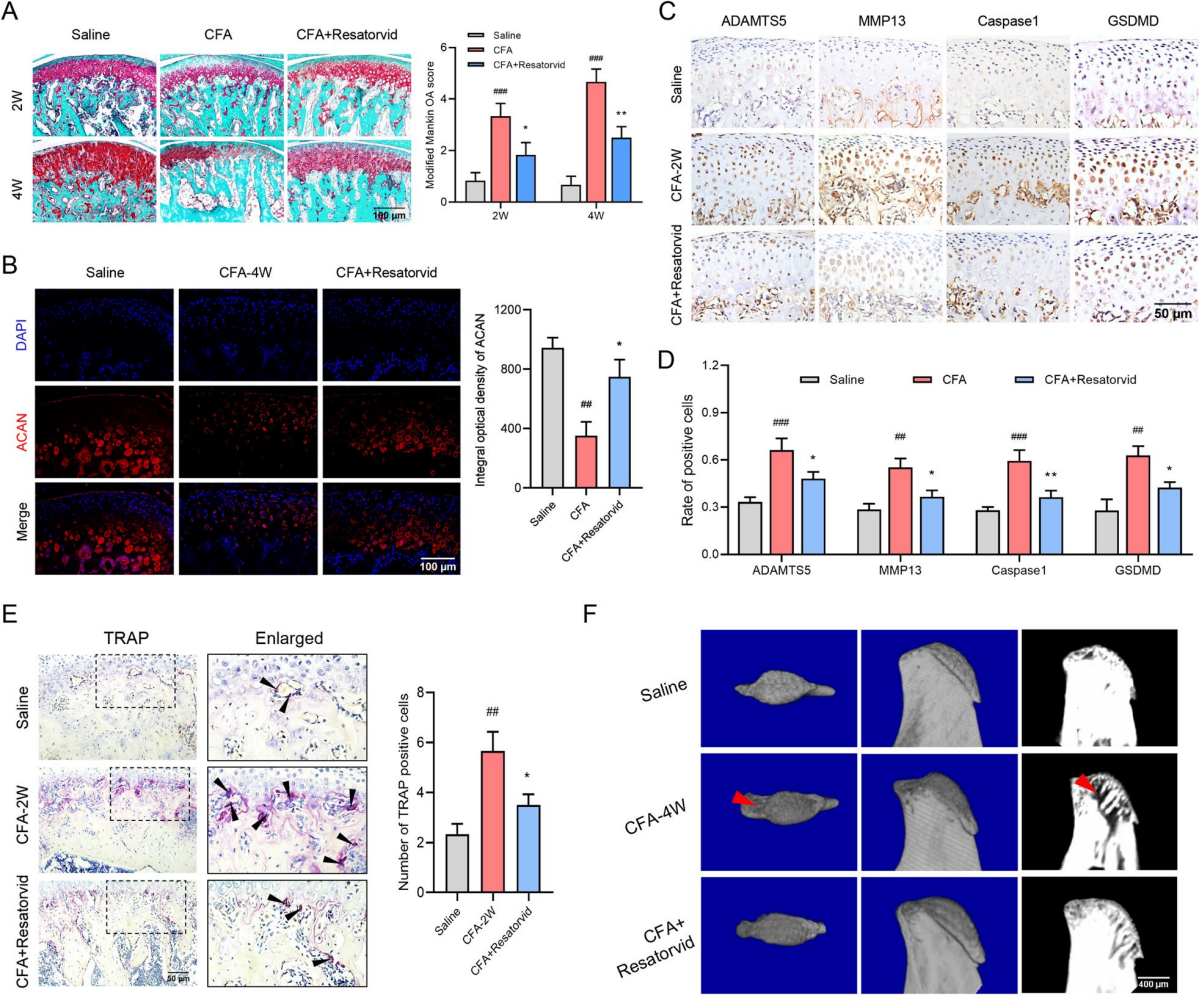
Systemic administration of Resatorvid mitigates cartilage degradation and bone destruction in CFA-induced TMJOA mice. **A** Safranin-O/Fast Green staining revealed significant loss of proteoglycans in CFA-induced TMJOA mice at 2 and 4 weeks, while intraperitoneal administration of Resatorvid alleviated CFA injection-induced proteoglycan loss. Quantitative analysis of the modified Mankin OA score verified the mitigative effect of Resatorvid administration on condylar cartilage degeneration in TMJOA mice, two-way ANOVA, mean ± SEM, *n* = 6 (###*p* < 0.001 *vs* saline group; *p < 0.05, **p < 0.01 vs CFA group). **B** Representative immunofluorescence images detected the production of aggrecan (ACAN) in the condylar cartilage of mice at 4 weeks and quantified by the integral optical density of ACAN, one-way ANOVA, mean ± SEM, *n* = 5 (##*p* < 0.01 vs saline group; *p < 0.05 *vs* CFA group). **C** Immunohistochemical staining was used to analyze the expression of cartilage degenerative mediators (ADAMTS5, MMP13) and pyroptotic biomarkers (Caspase-1, GSDMD) in the condylar cartilage of TMJOA mice 2 weeks after intra-articular CFA induction. **D** The rate of degenerative mediator- and pyroptotic biomarker-positive cells increased in the condylar cartilage of CFA-induced TMJOA mice, while intraperitoneal administration of Resatorvid downregulated the production of the mediators described above, one-way ANOVA, mean ± SEM, *n* = 6 (##*p* < 0.01, ###*p* < 0.001 vs saline group; *p < 0.05, **p < 0.01 vs CFA group). **E** Representative TRAP staining images of subchondral bone showed that increased osteoclast activity in TMJOA mice at 2 weeks was downregulated by systemic administration of Resatorvid, as quantified by the number of TRAP-positive cells, one-way ANOVA, mean ± SEM, *n* = 6 (##*p* < 0.01 vs saline group; *p < 0.05 vs CFA group). **F** Micro-CT analysis detecting bone destruction in TMJOA mice at 4 weeks, severe bone destruction and three-dimensional structural disorders in CFA-induced TMJOA mice, which were relieved in Resatorvid-treated TMJOA mice

Safranin-O/Fast Green and aggrecan (ACAN) immunofluorescence staining were utilized to examine cartilage degeneration in CFA-induced TMJOA mice. As depicted in Fig. 3A, remarkable proteoglycan loss occurred in the cartilage of CFA-induced TMJOA mice at 2 and 4 weeks, which was relieved by the administration of Resatorvid. The modified Mankin OA score was downregulated in Resatorvid-treated TMJOA mice, in contrast to CFA-induced TMJOA mice. Similar results were depicted by ACAN immunofluorescence staining of TMJOA mice at 4 weeks. Resatorvid treatment upregulated the production of ACAN in cartilage of CFA-induced TMJOA mice (Fig. 3B). Furthermore, immunohistochemical detection revealed that the positive staining of cartilage degenerative mediators (ADAMTS5, MMP13) and pyroptotic biomarkers (Caspase-1, GSDMD) was elevated in the condylar cartilage of TMJOA mice at 2 weeks but was reduced after the administration of Resatorvid (Fig. 3C and D).

The number of TRAP-positive cells (osteoclasts activated) in the subchondral bone was analyzed by TRAP staining. Activated osteoclasts were increased in CFA-induced TMJOA mice at 2 weeks and restrained in CFA + Resatorvid group mice (Fig. 3E). Micro-CT analysis displayed severe bone destruction and three-dimensional structural disorders in CFA-induced TMJOA mice at 4 weeks, which were reduced in Resatorvid-treated mice (Fig. 3F). These findings emphasized that prophylactic treatment with Resatorvid alleviates synovitis, cartilage degeneration and bone destruction in CFA-induced TMJOA mice.

### MyD88/NF-κB and NLRP3 signals are involved in the pathology of CFA-induced TMJOA mice

**Fig. 4:**
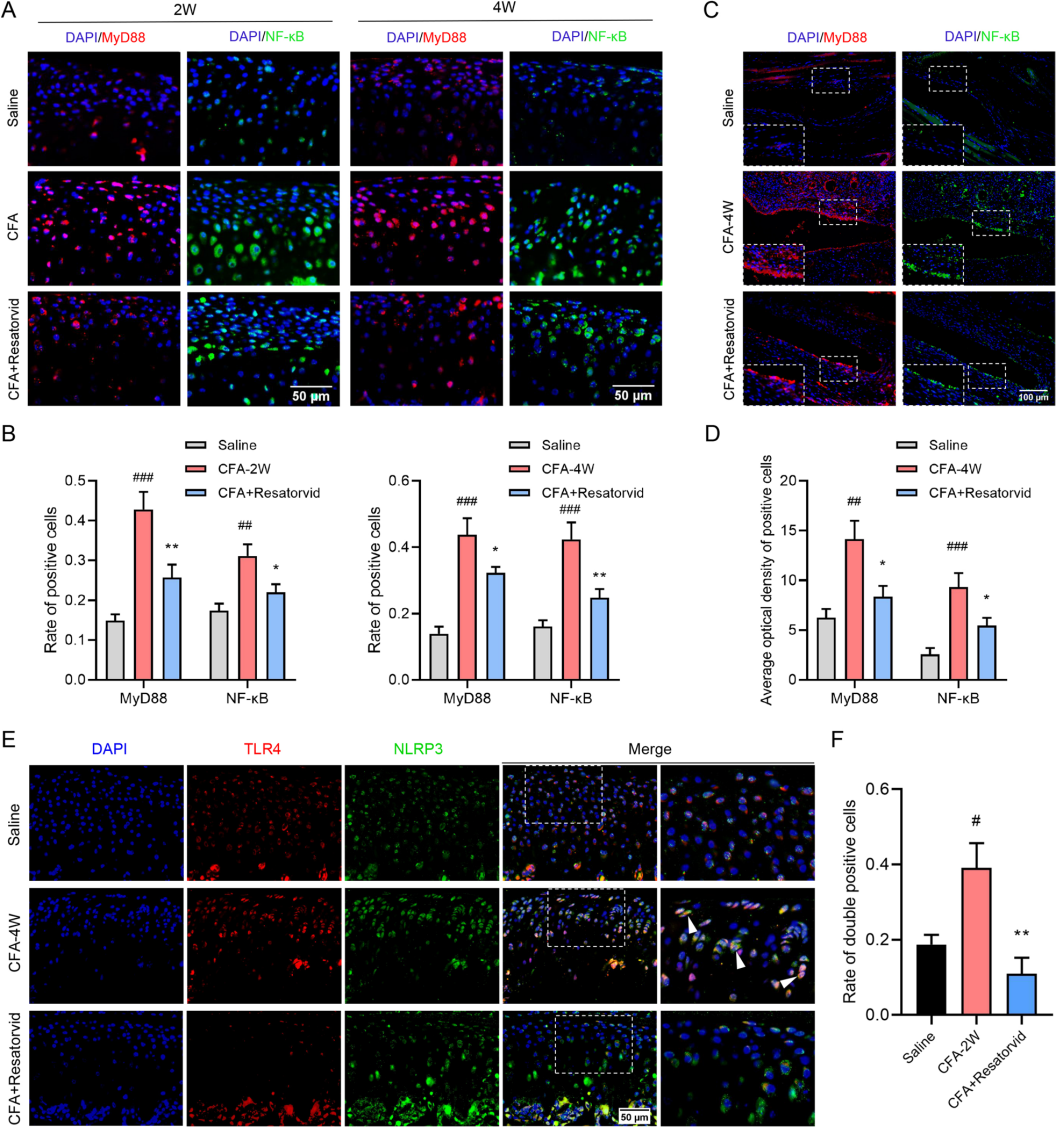
Resatorvid administration downregulates MyD88/NF-κB and NLRP3 signaling in the cartilage and synovium of CFA-induced TMJOA mice. **A** Representative immunofluorescence images analyzing the expression of MyD88 (red) and NF-κB (green) in the condylar cartilage of CFA-induced TMJOA mice at 2 and 4 weeks. **B** Quantitative analysis revealed that administration of Resatorvid reduced the rate of MyD88- and NF-κB-positive cells in the cartilage of CFA group mice at 2 and 4 weeks, respectively, one-way ANOVA, mean ± SEM, *n* = 6 (##*p* < 0.01, ###*p* < 0.001 vs saline group; *p < 0.05, **p < 0.01 vs CFA group). **C**, **D** Immunofluorescence staining detecting the production of MyD88 (red) and NF-κB (green) in the synovium of CFA-induced TMJOA mice at 4 weeks (**C**) and quantified by the average optical density of positive cells (**D**), one-way ANOVA, mean ± SEM, *n* = 6 (##*p* < 0.01, ###*p* < 0.001 vs saline group; *p < 0.05 vs CFA group). **E**, **F** Immunofluorescence double staining analyzing the coexpression of TLR4 (red) and NLRP3 (green) in the condylar cartilage of CFA-induced TMJOA mice at 4 weeks (**E**). The expression of TLR4 was significantly inhibited by Resatorvid administration, and the number of NLRP3-positive cells was also downregulated, as quantified by the rate of double-positive cells (**F**), one-way ANOVA, mean ± SEM, *n* = 5 (#*p* < 0.05 vs saline group; **p < 0.01 vs CFA group)

MyD88 and NF-κB have been accepted as vital downstream signals of TLR4 [28]. In the present study, the expression of MyD88 and NF-κB in condylar cartilage and synovial tissues of TMJOA mice was tested by immunofluorescence staining. As depicted in Fig. 4A, the positive staining of MyD88 and NF-κB was enhanced in the cartilage of CFA-induced TMJOA mice at 2 and 4 weeks and reduced significantly in Resatorvid-treated TMJOA mice (Fig. 4A and B). Similar incremental consequences were shown in the synovium of CFA-induced TMJOA mice at 4 weeks. Resatorvid treatment downregulated the expression of MyD88 and NF-κB in the synovium of TMJOA mice, as quantified by the AOD of positive cells (Fig. 4C and D). Immunofluorescence double staining of TLR4 and NLRP3 revealed significant inhibition of TLR4 and NLRP3 in the condylar cartilage of Resatorvid-treated TMJOA mice at 4 weeks, with the rate of TLR4 and NLRP3 double-positive cells downregulated compared with that in CFA-induced TMJOA mice (Fig. 4E and F). Collectively, these results suggest that MyD88/NF-κB and NLRP3 signals participate in synovitis and cartilage degeneration in CFA-induced TMJOA mice.

### Resatorvid treatment alleviates chondrocyte pyroptosis and degeneration through NLRP3 signaling

**Fig. 5:**
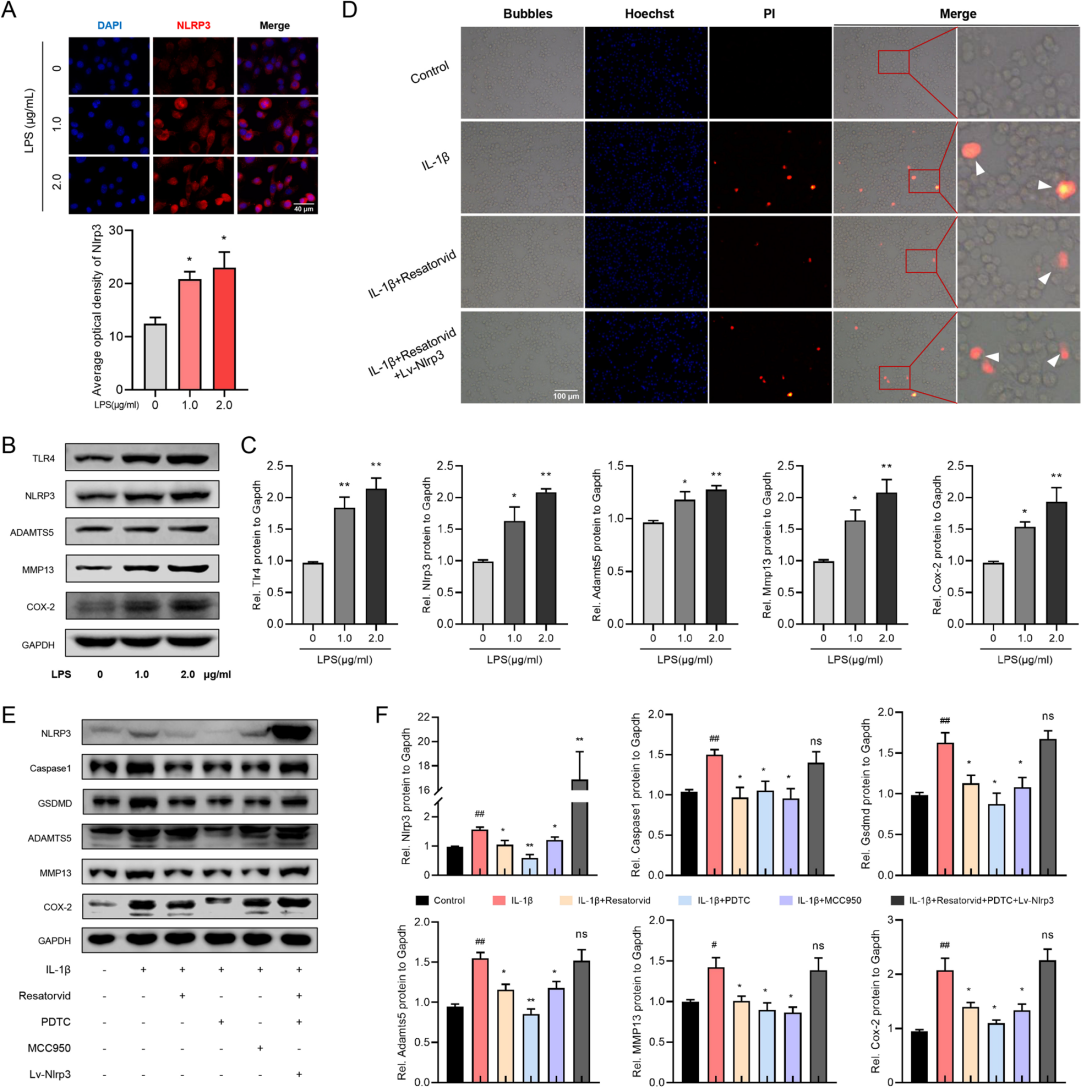
Resatorvid alleviates NLRP3-mediated chondrocyte pyroptosis and degeneration. **A** LPS was utilized as an activator of TLR4 and incubated with ATDC5 cells. Immunofluorescence detected that the expression of NLRP3 was enhanced in LPS-incubated ATDC5 cells in a dose-dependent manner, one-way ANOVA, mean ± SEM, *n* = 3 (*p < 0.05 vs 0 μg/ml). **B**, **C** Western blotting examining the effect of LPS stimulation on ATDC5 cells for 24 h (**A**). Quantitative analysis revealed that the production of TLR4, NLRP3, and degenerative mediators (ADAMTS5, MMP13 and COX-2) was increased in LPS-induced ATDC5 cells in a dose-dependent manner (**B**), one-way ANOVA, mean ± SEM, *n* = 3 (*p < 0.05, **p < 0.01 vs 0 μg/ml). **D** Phase-contrast and fluorescence images of ATDC5 cells stained with Hoechst (blue) and propidium iodide (PI, red, positive staining indicates lytic cell death). The pyroptotic cell bubbles (white arrows) in merged images revealed that Resatorvid treatment relieved IL-1β-irritated ATDC5 chondrocyte pyroptosis, which was reversed by NLRP3 overexpression. **E**, **F** Western blotting (**E**) and quantitative analysis (**F**) of the production of NLRP3, pyroptotic biomarkers (Caspase-1, GSDMD) and degenerative mediators (ADAMTS5, MMP13, COX-2) in ATDC5 cells treated as indicated. Treatment with Resatorvid, PDTC (NF-κB inhibitor), or MCC950 (NLRP3 inhibitor) downregulated the production of these mediators in IL-1β-induced ATDC5 cells, which was abrogated by overexpression of NLRP3, Student’s *t* test, mean ± SEM, *n* = 3 (#*p* < 0.05, ##*p* < 0.01 vs control group; *p < 0.05, **p < 0.01 vs IL-1β group, ns indicates no significant difference)

LPS was utilized as a TLR4 activator and was incubated in ATDC5 cells, which exhibited a dose-dependent elevation of NLRP3 by immunofluorescence staining (Fig. 5A). Moreover, western blotting analysis verified that the production of TLR4, NLRP3, and degenerative mediators (ADAMTS5, MMP13 and COX-2) was enhanced in TLR4-activated ATDC5 cells in a dose-dependent manner (Fig. 5B and C).

As shown in Fig. 5D, microscopy and PI staining for lytic cell death revealed that pyroptotic cell bubbles (white arrows in merged images) were increased in IL-1β-incubated ATDC5 cells and decreased by Resatorvid pretreatment. However, overexpression of NLRP3 abolished the protective effect of Resatorvid in IL-1β-irritated ATDC5 cells. Furthermore, western blotting revealed that the production of NLRP3, pyroptotic biomarkers (Caspase-1, GSDMD) and degenerative mediators (ADAMTS5, MMP13, COX-2) in IL-1β-incubated ATDC5 cells was downregulated by treatment with Resatorvid, PDTC (NF-κB inhibitor), or MCC-950 (NLRP3 inhibitor). Nevertheless, NLRP3 overexpression abrogated the suppressive effect of Resatorvid and PDTC on ATDC5 chondrocyte pyroptosis and degeneration (Fig. 5E and F). These findings indicated that Resatorvid treatment relieves chondrocyte pyroptosis and degradation through suppressing NLRP3 signaling.

### Resatorvid mitigates macrophage inflammation by inhibiting ROS production via NLRP3 signaling

**Fig. 6:**
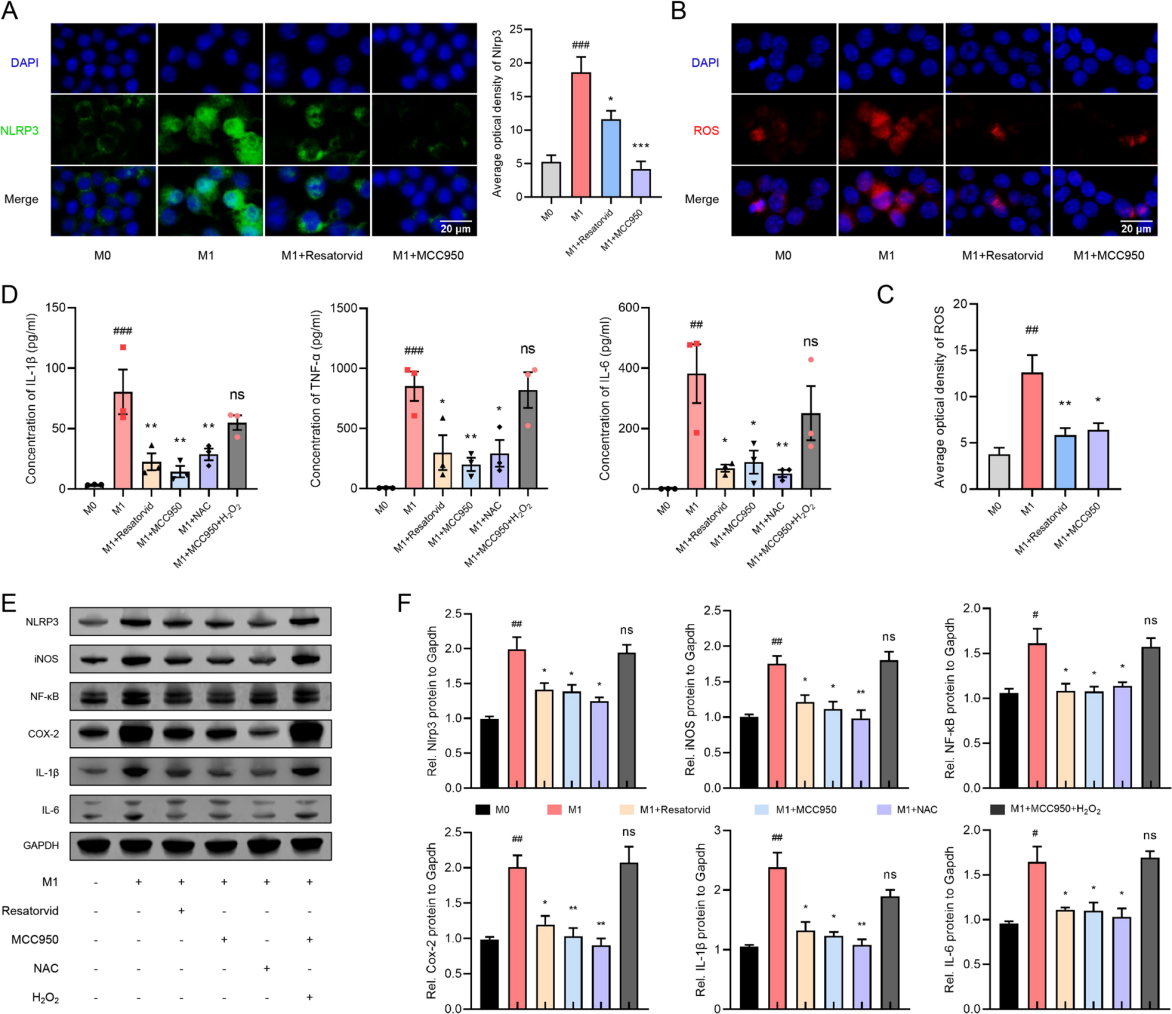
Resatorvid mitigates inflammatory responses of macrophages via NLRP3/ROS signaling. **A** Immunofluorescence and quantification of the expression of NLRP3 in Raw264.7 macrophages treated as indicated for 24 h. M1 macrophages were induced by 100 ng/ml LPS and 20 ng/ml INF-γ, one-way ANOVA, mean ± SEM, *n* = 3 (###*p* < 0.001 vs M0 group; *p < 0.05, ***p < 0.001 vs M1 group). **B**, **C** The production of ROS in Raw264.7 macrophages as specified was labeled by cell-permeable dihydroethidium (DHE) fluorogenic probes (**B**) and quantified by the average optical density of red fluorescence (**C**), one-way ANOVA, mean ± SEM, *n* = 3 (##*p* < 0.01 vs M0 group; *p < 0.05, **p < 0.01 vs M1 group). ELISA analysis detecting the concentration of cytokines (IL-1β, TNF-α, and IL-6) in the supernatant of Raw264.7 macrophages treated as indicated for 24 h, one-way ANOVA, mean ± SEM, *n* = 3 (##*p* < 0.01, ###*p* < 0.001 vs M0 group; *p < 0.05, **p < 0.01 vs M1 group, ns indicates no significant difference). **E**, **F** Western blotting analysis (**E**) and quantification (**F**) of the production of NLRP3 and proinflammatory factors (iNOS, NF-κB, COX-2, IL-1β, IL-6) in lysates of Raw264.7 macrophages treated as indicated, Student’s *t* test, mean ± SEM, *n* = 3 (#*p* < 0.05, ##*p* < 0.01 vs M0 group; *p < 0.05, **p < 0.01 vs M1 group, ns indicates no significant difference)

M1 macrophages from Raw264.7 cells were induced by LPS combined with INF-γ. The expression of NLRP3 was enhanced in M1 macrophages but suppressed by pretreatment with Resatorvid or MCC950 (Fig. 6A). Cell-permeable dihydroethidium (DHE) fluorogenic probes detected that the production of ROS was boosted in M1-polarized macrophages and downregulated by pretreatment with Resatorvid or MCC950 (Fig. 6B and C). As shown in Fig. 6D, the secreted proinflammatory cytokines (IL-1β, TNF-α, and IL-6) in the supernatant of Raw264.7 macrophages were significantly augmented in M1 macrophages but reduced by pretreatment with Resatorvid, MCC950, or NAC (ROS antagonist). Moreover, enrichment of ROS by H_2_O_2_ incubation reversed the inhibitory effect of MCC950 in M1 macrophages. This result indicated the potential downstream effects of ROS on NLRP3-mediated inflammatory responses of macrophages. Western blotting analysis verified that suppression of TLR4, NLRP3, or ROS by Resatorvid, MCC950, or NAC decreased the production of proinflammatory mediators (iNOS, NF-κB, COX-2, IL-1β, IL-6) in M1 macrophages, and incubation with H_2_O_2_ abolished the anti-inflammatory effect of MCC950 in M1 macrophages (Fig. 6E and F). Collectively, these findings indicate that Resatorvid mitigates macrophage inflammation by inhibiting ROS production via NLRP3 signaling.

## Discussion

The latest theories of OA pathogenesis implicate the activation of the innate immune system as intricately involved in the initiation and perpetuation of articular inflammation, and increasing attention has been given to the role of innate immunity mediated by inflammatory responses during OA pathology [5, 29]. The pathogenesis of TMJOA emphasizes the interplay between excessive mechanical loading and chronic inflammation [1]. In this study, we are devoted to researching the regulatory role of Resatorvid on experimental inflammation-induced TMJOA pathology. There was conspicuous synovial inflammation (including synovial inflammatory exudation, inflammatory cell infiltration, lipid droplet and lymphoid follicle formation, and lining layer thickening), gradual cartilage degeneration, and bone destruction in CFA-induced TMJOA mice, accompanied by elevated expression of TLR4 in the synovium and condylar cartilage. Prophylactic administration of Resatorvid by intraperitoneal injection ameliorated synovitis, cartilage degradation, and bone destruction in CFA-induced TMJOA mice. Histological detection showed reduced macrophage activation and cytokine secretion in synovial tissues, downregulated degenerative mediators and pyroptotic biomarkers in cartilage, and alleviated osteoclast activation in subchondral bone of Resatorvid-treated TMJOA mice. The findings of this study regarding the protective effect of Resatorvid in an experimental inflammatory TMJOA model have not been documented but have been reported in knee OA and rheumatoid arthritis (RA) pathogenesis. Jiang’s study verified that administration of Resatorvid suppressed cartilage degeneration and inflammatory and immune responses in a rat OA model [30]. Samarpita et al. confirmed that Resatorvid significantly reversed the body weight and paw thickness of adjuvant-induced arthritis (AIA) rats to the normal state, reduced the increased serum inflammatory factor levels, and ameliorated joint inflammatory symptoms [31].

The NLRP3 inflammasome has been confirmed to be closely connected with inflammation-induced pyroptosis and oxidative stress in various inflammatory diseases [32–34]. NLRP3-mediated chondrocyte pyroptosis has been accepted as a type of cellular death during OA pathogenesis [10, 35]. Bai et al. discovered that activation of the adenosine A3 receptor attenuates the progression of OA by inhibiting ROS production and NLRP3-induced chondrocyte pyroptosis [36]. Furthermore, the regulation of the TLR4/NF-κB signaling pathway in NLRP3 inflammasome-induced pyroptosis during OA has been verified [37]. However, the regulatory function of NLRP3 in condylar chondrocyte pyroptosis during TMJOA is still unknown. Herein, not only the expression of TLR4 downstream signals (MyD88 and NF-κB) but also NLRP3 signaling was downregulated in the synovium and cartilage of Resatorvid-treated TMJOA mice in this study, indicating that NLRP3 potentially acts as a downstream signaling of TLR4. Synovitis and cartilage degeneration are the primary pathologic changes in TMJOA, suggesting potential therapeutic targets. Numerous studies have explored the potential role of synovial macrophage inflammation and cartilage metabolic disorders in TMJOA [9, 38–40]. In the present study, we confirmed the downregulated activation of synovial macrophages and metabolic disorder of condylar cartilage in Resatorvid-treated TMJOA mice, indicating the potential protective effect of Resatorvid in macrophage activation and cartilage disorders during TMJOA pathology.

Ulteriorly, an ex vivo study found that activation of TLR4 by LPS incubation in ATDC5 chondrocytes enhanced the production of NLRP3 and degenerative mediators, while Resatorvid treatment or NLRP3 inhibition suppressed IL-1β-induced pyroptosis and degeneration of chondrocytes. Furthermore, upregulation of NLRP3, ROS, and proinflammatory cytokines was discovered in M1 macrophages, which was reversed by Resatorvid treatment or NLRP3 inhibition. Similarly, Luo et al. verified the crucial negative regulatory effect of IL-37 and IL-38 on NLRP3-mediated M1 macrophage activation and chondrocyte inflammation, respectively [41, 42]. These results indicate the vital regulatory role of NLRP3 in synovial macrophage activation and chondrocyte degeneration during TMJOA pathology. Collectively, these findings emphasize the mitigative effect of Resatorvid on chondrocyte pyroptosis and macrophage oxidative stress during chondrocyte degeneration and macrophage inflammation, respectively, via NLRP3 signaling.

Certain deficiencies still exist in the current study. First, although systemic administration of Resatorvid by intraperitoneal injection mitigated synovial inflammation and cartilage degeneration in a highly inflammatory TMJOA animal model, the potential oral medication effect for TMJOA needs further verification concerning its further clinical application. Second, primary cells could better illustrate chondrocyte pyroptosis and synovial macrophage inflammation, whereas they are easily intended to lose their phenotypes during two-dimensional culture [43], leading to the widespread utilization of ATDC5 and RAW264.7 cells as eligible alternatives [44–46]. Last, the alleviating effect of Resatorvid on the oxidative stress of macrophages in vivo still needs to be further verified.

## Conclusions

**Fig. 7:**
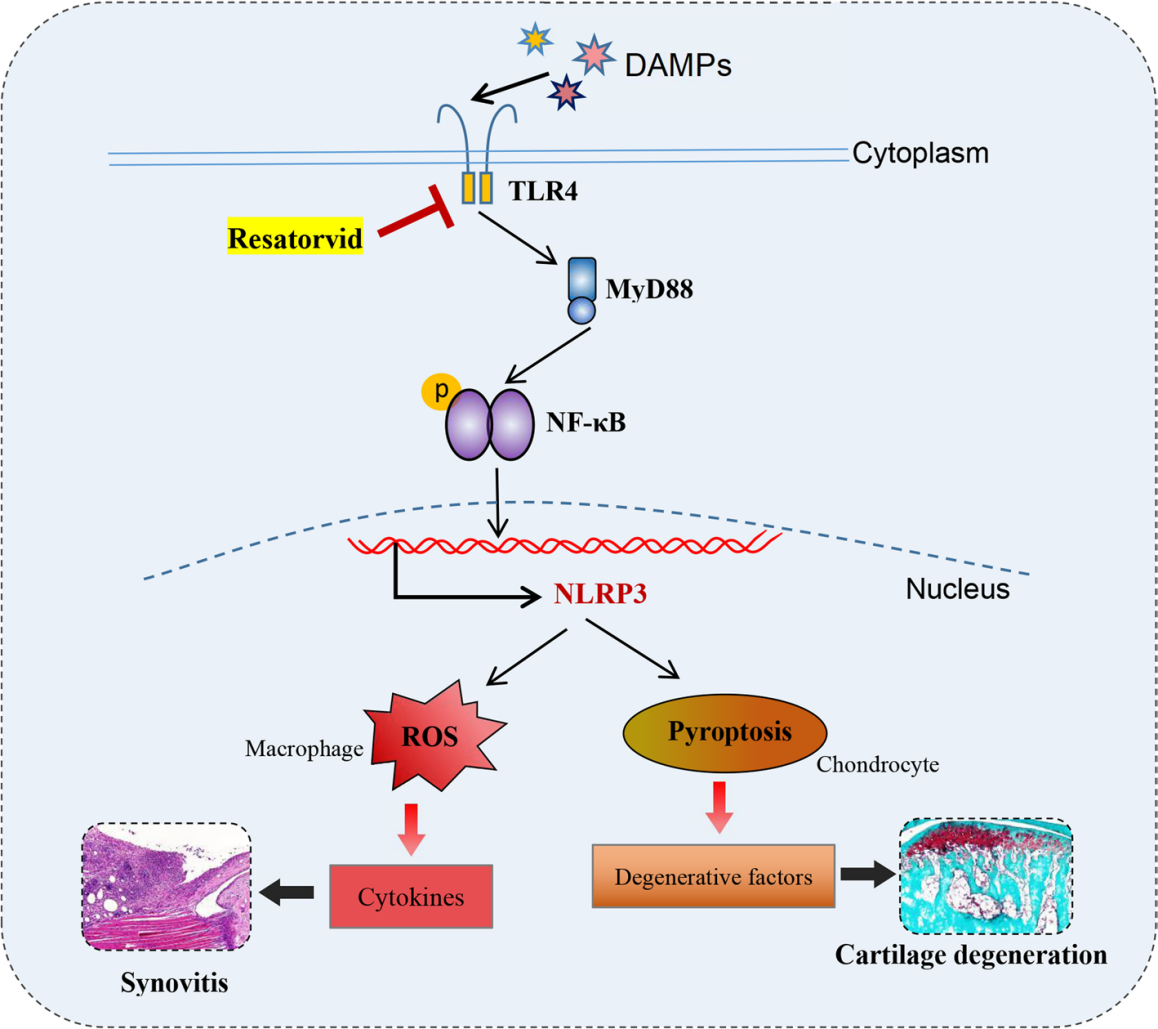
Schematic diagram of Resatorvid on cartilage degradation and synovitis during TMJOA. Resatorvid mitigates synovial inflammation and cartilage degradation during TMJOA through alleviating ROS-mediated proinflammatory cytokine secretion from macrophages, pyroptosis, and chondrocyte degeneration via the TLR4/MyD88/NF-κB/NLRP3 signaling pathway

Taken together, the results of this study show that prophylactic treatment with Resatorvid alleviates synovial inflammation and cartilage degradation in CFA-induced TMJOA mice by suppressing ROS-induced proinflammatory cytokine secretion by macrophages as well as chondrocyte pyroptosis and degeneration through the TLR4/MyD88/NF-κB/NLRP3 signaling pathway (Fig. 7).

### Supplementary Information

**Additional file 1. **Uncropped gel and blot images.

## Data Availability

The data supporting the findings are presented in the article or further requested from the corresponding author under reasonable necessity.

## Abbreviations

TMJOA: Temporomandibular joint osteoarthritis
TLR4: Toll-like receptor 4
CFA: Complete Freund’s adjuvant
NLRP3: NOD-like receptor protein 3
ROS: Reactive oxygen species
TMD: Temporomandibular disorder
PRRs: Pattern recognition receptors
OA: Osteoarthritis
ECM: Extracellular matrix
GSDMD: Gasdermin D
TMJ: Temporomandibular joint
EDTA: Ethylene diamine tetra-acetic acid
AOD: Average optical density
IOD: Integral optical density
ITS: Insulin-transferrin-selenium
FBS: Fetal bovine serum
BSA: Bovine serum albumin
DAPI: 4′,6-Diamidino-2-phenylindole
PI: Propidium iodide
DHE: Dihydroethidium
ACAN: Aggrecan
RA: Rheumatoid arthritis
AIA: Adjuvant-induced arthritis
