# Supplementary figures and images for: Resatorvid alleviates experimental inflammatory TMJOA by restraining chondrocyte pyroptosis and synovial inflammation
Source: Arthritis Res Ther. 2023 Nov 29;25:230. doi: 10.1186/s13075-023-03214-4 (PMC10685467; doi:10.1186/s13075-023-03214-4)

**Figure 5B**

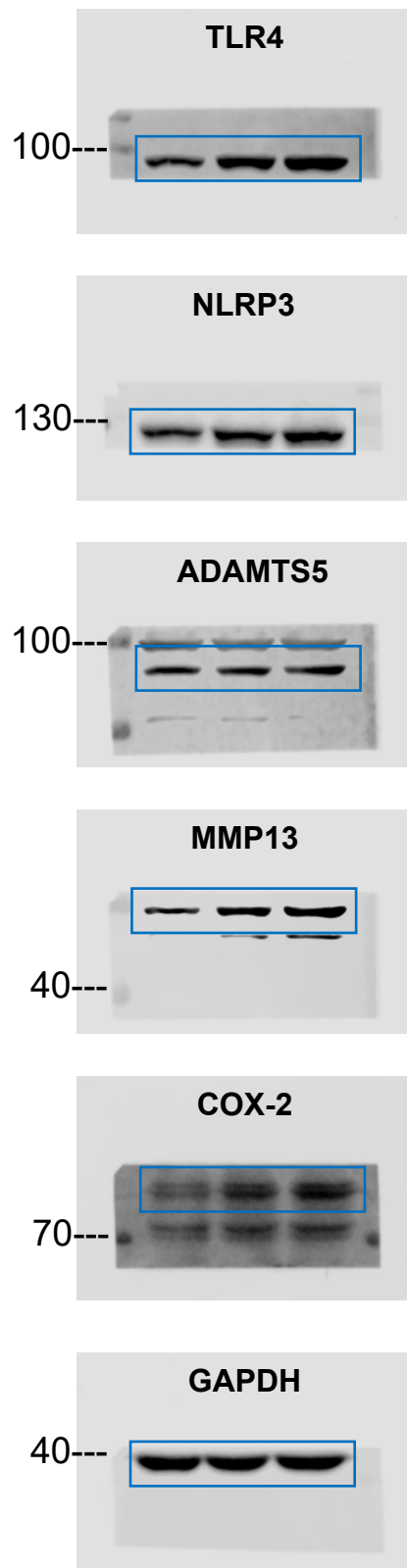

**Figure 5E**

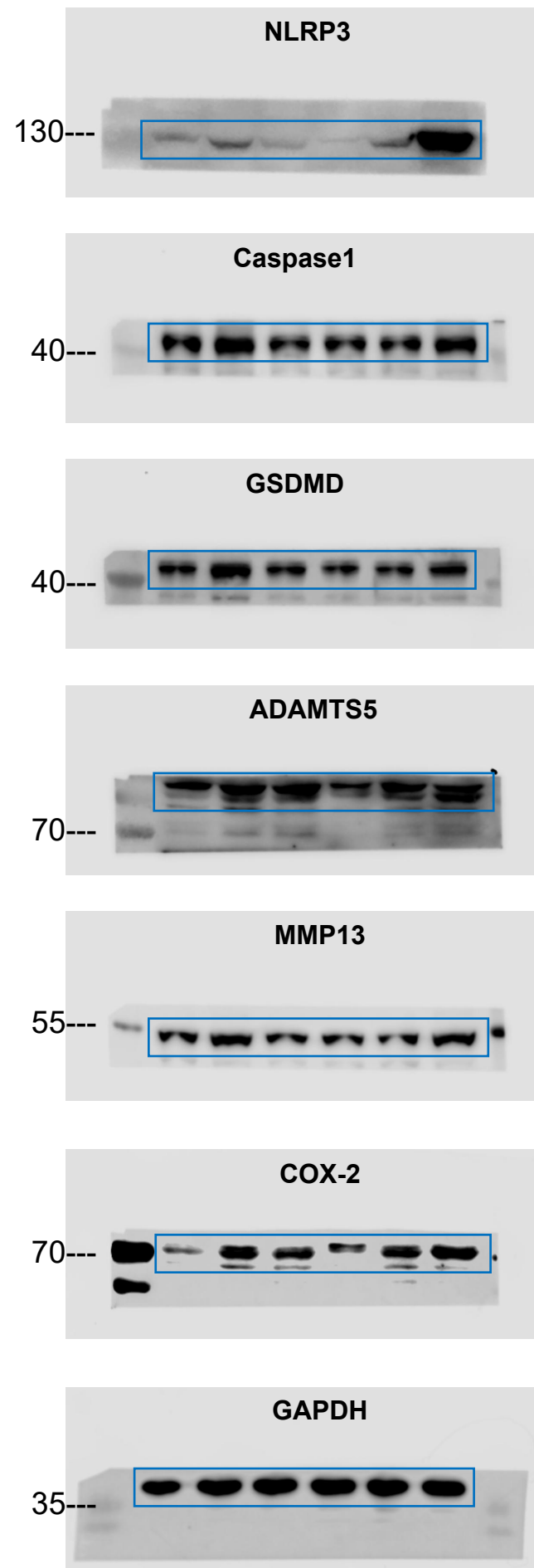

**Figure 6E**

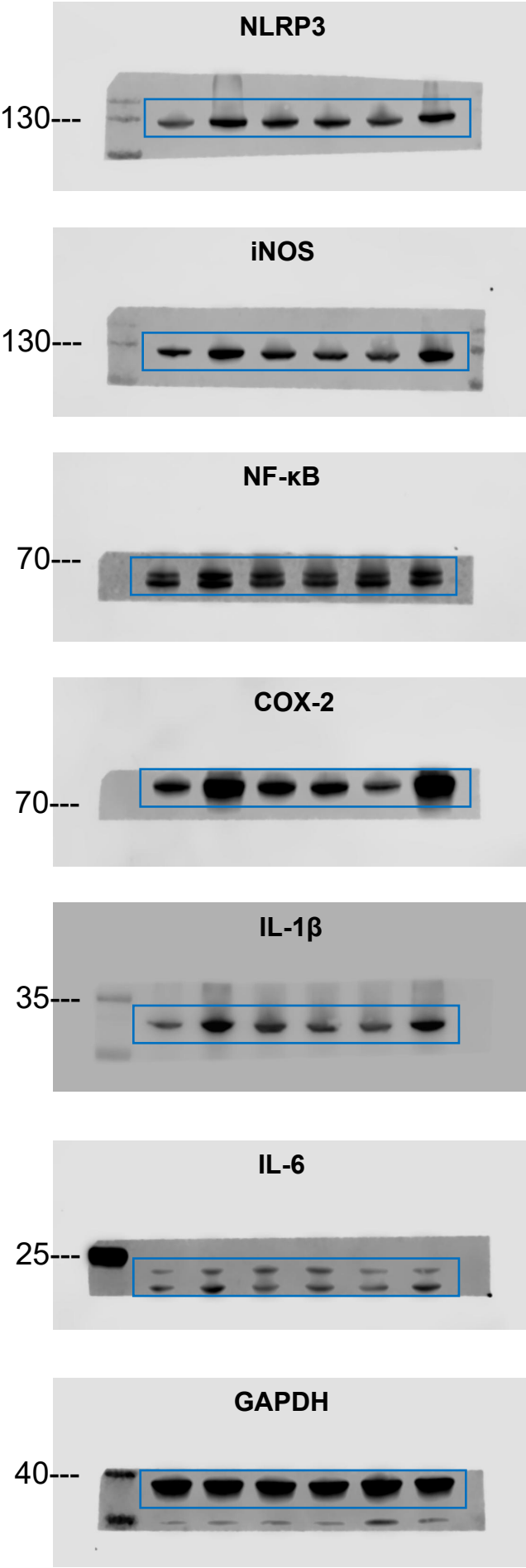

Supplement: Supplementary file 1 — Additional file 1. Uncropped gel and blot images. [file 13075_2023_3214_MOESM1_ESM.pdf]
